# A Novel Smart Contract Vulnerability Detection Method Based on Information Graph and Ensemble Learning

**DOI:** 10.3390/s22093581

**Published:** 2022-05-08

**Authors:** Lejun Zhang, Jinlong Wang, Weizheng Wang, Zilong Jin, Chunhui Zhao, Zhennao Cai, Huiling Chen

**Affiliations:** 1 College of Information Engineering, Yangzhou University, Yangzhou 225127, China; mz120200903@yzu.edu.cn; 2 Research and Development Center for E-Learning, Ministry of Education, Beijing 100039, China; 3 Cyberspace Institute Advanced Technology, Guangzhou University, Guangzhou 510006, China; 4 Computer Science Department, City University of Hong Kong, Kowloon Tong, Hong Kong; m5232117@u-aizu.ac.jp; 5 School of Computer and Software, Nanjing University of Information Science and Technology, Nanjing 210044, China; zljin@nuist.edu.cn; 6 College of Computer Science and Technology, Harbin Engineering University, Harbin 150001, China; zhaochunhui@hrbeu.edu.cn; 7 Department of Computer Science and Artificial Intelligence, Wenzhou University, Wenzhou 325035, China; cznao@wzu.edu.cn

**Keywords:** smart contract, vulnerability detection, blockchain security, operation flow, Ensemble Learning, information graph

## Abstract

Blockchain presents a chance to address the security and privacy issues of the Internet of Things; however, blockchain itself has certain security issues. How to accurately identify smart contract vulnerabilities is one of the key issues at hand. Most existing methods require large-scale data support to avoid overfitting; machine learning (ML) models trained on small-scale vulnerability data are often difficult to produce satisfactory results in smart contract vulnerability prediction. However, in the real world, collecting contractual vulnerability data requires huge human and time costs. To alleviate these problems, this paper proposed an ensemble learning (EL)-based contract vulnerability prediction method, which is based on seven different neural networks using contract vulnerability data for contract-level vulnerability detection. Seven neural network (NN) models were first pretrained using an information graph (IG) consisting of source datasets, which then were integrated into an ensemble model called Smart Contract Vulnerability Detection method based on Information Graph and Ensemble Learning (SCVDIE). The effectiveness of the SCVDIE model was verified using a target dataset composed of IG, and then its performances were compared with static tools and seven independent data-driven methods. The verification and comparison results show that the proposed SCVDIE method has higher accuracy and robustness than other data-driven methods in the target task of predicting smart contract vulnerabilities.

## 1. Introduction

The recent advances in information and communication technology (ICT) have promoted the evolution of the conventional computer-aided industry into the smart industry [1]. In the transformation, the Internet of Things (IoT) has an essential role in linking the physical industrial space and cyberspace. However, the current IoT is not well-suited to the needs of the industry in terms of privacy and security. Thanks to the continuous progress of blockchain technology, the combination of IoT and blockchain is becoming more and more popular among security personnel. For example, the study [2] provides a worthwhile research solution for data privacy transfers in the security domain. Yet blockchain still is experiencing security issues of its own while solving IoT privacy and security problems. Currently, the security of smart contracts has become one of the key blockchain security issues.

Smart contracts in blockchain are automated, programable, verifiable, and open and transparent. Using these features of smart contracts transforms them into programed, executable, and verifiable ways for decentralized execution, thereby significantly reducing the underhanded operations and unfair practices that exist in decentralized systems. More and more individual developers or industry practitioners can develop Decentralized Applications (DApps) [3], such as Decentralized Finance (DeFi), etc. In the metaverse, users can also co-edit the world, realize the value exchange through smart contracts, and guarantee the transparent enforcement of system rules. However, previous research has shown that many real-world smart contracts deployed on blockchains have serious vulnerabilities [3], for example, the DAO attack [4] and the Parity attack [5]. The DAO attack exploits a recursive call vulnerability to transfer one third of the DAO funds to a malicious account (worth about USD 50 million). The Parity attack exploits a vulnerability in the library contract to steal over 150,000 ETH to a malicious account (worth about USD 30 million).

Recent studies have reported many methods for smart contract vulnerability prediction. In general, these methods can be classified into two categories: (1) expert rule-based approach, considering static symbolic analysis [6,7], dynamic symbolic execution [8], combined dynamic and static methods [9], and XPath [10]; and (2) data-driven approach, i.e., using NN approach [11,12,13,14,15,16,17].

In symbolic-execution-based approaches, symbolic execution models are often used together with experimental data to accomplish the task of contract vulnerability prediction. In the last few years, many successes have been achieved using symbol-execution-based methods. However, constructing an accurate symbolic execution model is not an easy task, as it often requires extensive knowledge of Solidity programing (Solidity is used as an example in this paper) or experimental data obtained under well-designed and controlled conditions, which are often untimely or too time-consuming.

As an alternative to expert rule-based methods, data-driven methods for smart contract vulnerability prediction have gained popularity in recent years. The data-driven approach relies entirely on the data themself and usually does not require much knowledge of how the data work exactly. In the data-driven approach to contract vulnerability prediction, contract vulnerability prediction can be performed by learning complex features extracted from the operational sequence dependencies of a contract, and this dependency can be learned with various ML methods. The focus of the work in this paper is to apply different NNs to contractual vulnerability prediction. NN is a structure consisting of multiple layers of interconnected “neurons” that map input features to outputs (such as the vulnerability labels in this paper) [12,13,14,15,16]. NN-based deep learning methods have recently attracted the attention of researchers working in cybersecurity because of their ability to automatically extract features and their good generalization performance. Huang et al. [11] designed a multi-task NN model based on an attention mechanism. The model uses a shared embedding layer with the sequence of operations of a smart contract as its input, and each subtask uses the shared embedding features. The effectiveness of the proposed NN approach in vulnerability prediction is also verified on three vulnerabilities. Recently, Hongjun Wu et al. [17] proposed a model using key data flow graphs and pretraining techniques for smart contract vulnerability prediction based on code graphs. The method converts the smart contract source code into an abstract syntax tree (AST), finds the locations and relationships of variables in the contract, and constructs a code graph for verifying the model performance by using the variables associated with vulnerabilities as nodes.

Although these data-driven methods can provide satisfactory accuracy in vulnerability prediction, they focus on improving the underlying model or single accuracy and need to be backed by a large-scale training dataset. At the same time, the time and human costs of collecting data are rarely mentioned in the studies of these methods. In many real-world scenarios, collecting contract vulnerability data is an expensive and time-consuming process. To alleviate this problem, this paper proposes an EL-based vulnerability prediction approach, called SCVDIE-ENSEMBLE, which is based on seven different neural networks using contract vulnerability data for contract-level vulnerability detection. The accuracy and robustness of the proposed method can be improved by using EL, where the final decision is reached by weighting the individual predictions (i.e., the vulnerability prediction labels of a single NN model).

The proposed SCVDIE-ENSEMBLE approach consists of four main steps, which are outlined below.

First, n-fold cross-validation (CV) is performed using the source dataset, including the original contract data collected from DEDAUB [18], to obtain 7 ∗ n pretraining results, which will help create more diverse NN models and their parameters. The diversity of these independent models comes from the different training subsets used for pretraining, where each independent NN model focuses on fitting a different sub-sample of the source dataset.

Second, a multi-layer perceptron is used to combine the vulnerability prediction results of individual NN models. This approach causes the good predictions to be amplified and the bad predictions to be attenuated.

The experimental results show that the proposed SCVDIE-ENSEMBLE method can provide more accurate and robust vulnerability prediction. Compared to some other ML methods including each NN model, the proposed SCVDIE-ENSEMBLE method can provide more accurate vulnerability predictions using a relatively small training dataset. To the best of our knowledge, this is the first attempt to use EL methods on smart contract vulnerabilities to improve the performance of deep learning in predicting contract vulnerabilities from operational sequence data.

In this study, several contributions were made, as detailed below.

First, this paper proposes the SCVDIE-ENSEMBLE model for predicting the possibility that smart contracts contain vulnerabilities, and their source code is public [19]. The experimental results in this paper show that: (1) SCVDIE-ENSEMBLE outperforms other ML methods; and (2) EL can improve the robustness of SCVDIE-ENSEMBLE models.

Second, the effectiveness of the proposed SCVDIE-ENSEMBLE model in making accurate vulnerability predictions is based on more than 21,667 smart contracts manually collected and tagged from DEDAUB, of which 11,756 are vulnerable smart contracts and 9911 are nonvulnerable smart contracts; this process took more than 2 weeks.

Third, in this paper, the performance of eight deep learning models is experimentally demonstrated by using different size datasets for training. The conclusions show that integrating EL can not only reduce the size of the training dataset with the same error of the model, but also effectively improve the accuracy and robustness of vulnerability prediction.

The remainder of this paper is structured as follows: Section 2 reviews the existing studies, including conventional methods, general ML methods, and software bug analysis research applying EL. In Section 4, the research design of the experiments in this paper is described. The way of building the dataset is also introduced. In Section 5, the experiments and results of SCVDIE are presented, and the conclusions of the analysis. In Section 6, SCVDIE’s characteristics and limitations are analyzed. In Section 7, the conclusion of this paper and future work is discussed.

## 2. Related Work

The successes of neural techniques in many areas motivated researchers to apply neural networks for code analysis for the detection of software defects and vulnerabilities [20]. This section will review the existing methods, including conventional methods, neural-network-based smart contract vulnerability detection technologies, and EL technologies.

### 2.1. Conventional Methods

Oyente [21] simulates the execution of the entire program by constructing a control flow graph, solving the constraints using the z3 constraint solver, and determining the jump direction of the program blocks based on the solution results. As one of the first studies to use symbolic execution to detect smart contract source code or bytecode vulnerabilities, Oyente provides a symbolic execution interface to other static scanning tools. Similarly, Mythril [22], Manticore [23], etc., are analyzed based on symbolic execution. Another common analysis method is formal verification. Hirai et al. [24] verify the security of contracts by formalizing them. In addition, ZEUS [25] and Securify [26] are also two common formal verification tools.

### 2.2. Machine Learning

As mentioned in the introduction, security problems in smart contracts have caused huge economic losses, and more and more security researchers are using ML for contract vulnerability detection, with many fruitful results. These studies [27,28] used the GNN to normalize the graph constructed by smart contracts and use the graph to extract features. These studies [29,30] constructed the AST of smart contracts, and then extracted features from the perspective of AST. The study [31] used static and dynamic methods for vulnerability detection, used Word2Vec [32] for embedding, and used logistic regression for feature extraction. To detect unknown types of vulnerabilities, dynamic fuzzing methods were also introduced. The study [33] used the Average Stochastic Gradient Descent Weighted Dropout Long Short-Term Memory (AWD-LSTM) Model, replacing the “decoder” layer of AWD-LSTM with a fully connected layer. This approach is better than random initialization because the network already contains a lot of semantic information about the input data. The study [3] used the pretrained Bert model to process the smart contract source code but did not consider the semantic and grammatical connections between functions. This method uses the contract source code as a text sequence, each code element as a token, and then performs token embedding, segment embedding, and position embedding separately. After the embedding is completed, the result is predicted by training the classifier.

### 2.3. Ensemble Learning

EL is a learning method in which predictions given by different learning models are aggregated to arrive at a possibly optimal final decision [34]. Its main purpose is to decrease the peril of choosing a single model with poor performance and to improve the performance of one model by using an intelligent ensemble of several separate models [34,35]. In ML, when the test set and training set obey different distributions, there will be a large deviation in the prediction results if the original training model is used. A common approach to this problem is to recollect training sets and test sets with the same or similar distribution and train new models. Nevertheless, recollecting enough contract data to rebuild a model is expensive on actual blockchains, and it is not even possible to have so many samples to choose from. Efforts to try to solve the challenge have promoted the progression of EL, which has better strategies for datasets of all sizes. When the dataset is small, using the bootstrap [36] method for sampling can obtain multiple datasets, train multiple models separately, and then combine them, which are sufficient to reconstruct an ML model. For this reason, EL has become a popular learning structure. Image classification [37,38] is one of its classic applications. The financial crisis [39,40] is one of the applications of EL in real life. Further, EL plays a crucial role in economic decision making. Moreover, EL has been used in computer-assisted medicine and 5G-based telemedicine successfully [41,42]. It can also be used for various types of program vulnerability detection to predict the probability that the program contains vulnerabilities [43,44]. However, exploiting EL for smart contract vulnerability prediction is still in its infancy. As of this writing, this study is the first attempt to apply EL to blockchain smart contract vulnerability prediction.

## 3. Methodology

In this subsection, the design and implementation of SCVDIE are presented, as well as a novel dataset constructed that can be used to build an integrated system for smart contract vulnerability detection by using the NN model. SCVDIE analyzes COS (i.e., critical opcodes sequences directly related to the vulnerability) from the source code and tries to extract vulnerable opcode fragments. Then, vulnerability identification is designed as a classification task, where each sequence is assigned a vulnerability probability. Sequences with higher probability will be considered fragile. With enough training, SCVDIE can learn the possible patterns of vulnerabilities, as reflected by the different parameters of the model, i.e., the diversity of parameters. These different parameters are applied to samples with similar characteristics not seen in the model, and predictions are made accordingly.

### 3.1. Overall Architecture

**Figure 1:**
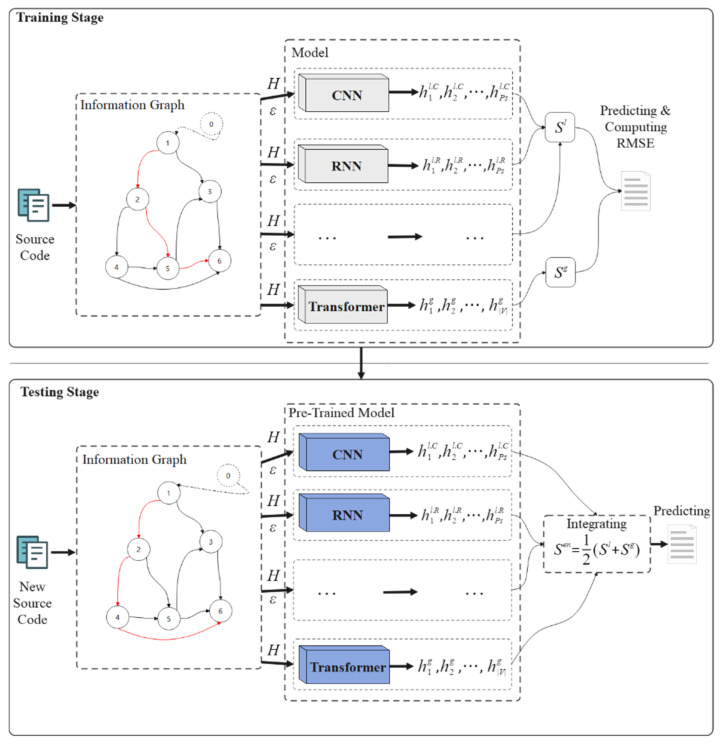
SCVDIE overview.

Identifying smart contract vulnerabilities can be seen as classifying the corresponding opcodes into vulnerable/invulnerable sequences. Therefore, the problem of identifying vulnerable smart contracts is defined as identifying vulnerable operations. Figure 1 illustrates the overall design of the proposed SCVDIE in this paper. Both the training and testing phases consist of three main steps: constructing IGs from co-occurrence frequencies, embedding IGs into the matrix space, training or predicting them, and aggregating the results of different NN models to output a final decision. Before starting to present the specific structure of SCVDIE, it is very critical to provide a global description of the notation used in this paper to enhance its readability, as shown in Table 1.

### 3.2. Building Vectorized IGs

#### 3.2.1. Determine the Same (Co-) Present Relationship

**Figure 2:**
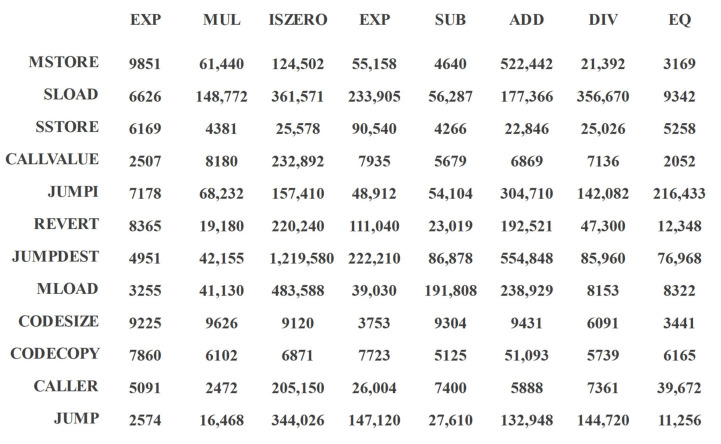
Co-occurrence frequency.

The frequency of co-occurrences was calculated from the opcodes of all contract samples in this paper, where the opcodes of individual smart contracts can be counted from the Solidity compiler. Figure 2 shows the co-occurrence frequency matrix of the 20 operands with the highest co-occurrence frequency.

Taking MSTORE in the first row as an example, it can be seen that the vertical co-ordinate with the highest frequency corresponds to ISZERO, and the lowest frequency corresponds to EQ. In building IGs, the ISZERO node is the closest to MSTORE, and EQ and MSTORE have the farthest distance from each other. The operands that belong to the same horizontal or vertical co-ordinate are ordered according to the order of their appearance in the contract.

#### 3.2.2. Building Graph

The purpose of this work is to evaluate the vulnerability of smart contracts. SCVDIE is pretrained in a supervised learning environment, the tags of smart contracts are initially labeled by static recognition tools, potential COS are identified by predefined rules, and, finally, COS is used as input data to construct an infographic IG based on co-occurrence relations to represent the vulnerability pattern information of the program (the infographic part in Figure 1). The nodes of IG are composed of opcodes, and all nodes are connected according to the position–frequency relationship, and opcodes belonging to the same class are connected in the order of their occurrence so that all edges and nodes form a unilateral connected digraph (UCD). This paper considers that IG consists of a set of nodes and undirected edges, denoted $IG=\left(V,E\right)$, where V denotes the set of nodes of IG and E represents the set of edges connecting these nodes. Given an infographic IG, one goal of the vulnerability identification task is to uncover hidden vulnerability features by identifying the connection patterns between nodes. Therefore, this paper constructed the samples in the dataset as $D=\left\{IG_{i},y_{i}\right\}$, where 1≤i≤N, $y_{i}$ denote the label of the ith sample, $\left|V\right|$ denotes the number of nodes of $IG_{i}$, and N is the original number of samples in the experimental dataset.

#### 3.2.3. Graph Vectorization

When plotting these embedding matrices in a vector space, the original input code sequence is transformed into a meaningful matrix where nodes with similar relationships are located very closely together, which allows the neural network to learn from rich relationships. To vectorize the node content into a matrix, this paper first constructed the IG of all sample contracts, and then extracted all paths of each IG. Based on these paths, a set containing many operation sequences was built, and, finally, embedding models such as Word2vec were trained with the set. For each path, this paper concatenated the token embeddings corresponding to the path nodes as the initial representation of the path. Just like human language, the same word has different meanings in different contexts. A smart contract contains multiple functions, and the same operation may have different results in different functions, so this paper designed a novel node embedding method F to vectorize the node $v_{i}$ into $\beta_{i}\in {\mathbb{R}}^{d}$, where d is the embedding dimension. The $\beta_{i}$ contains all the embedded nodes of the graph and is defined as B. Given a sample $\left\{IG,y\right\}$, then input the vectorized graph into a neural network model to learn node information representation $B^{\prime }=F_{model}(B,E)$. A good model can fully embed the semantic information of nodes and their contexts, so this paper input the semantic embedding $\beta^{\prime }$ corresponding to each node into the classifier, and then calculated the vulnerability probability of each node. More precisely, the learned $B^{\prime }\in {\mathbb{R}}^{d_{h}\times \left|V\right|}$ were fed into a linear layer with trainable weights $W\in {\mathbb{R}}^{d_{h}\times 1}$ and biases $b={\mathbb{R}}^{1\times \left|V\right|}$ to obtain a score for each node, where $d_{h}$ is the hidden dimension of the model, followed by a softmax layer with probability $P=softmax\left(W^{T}B^{\prime }+b\right)$. Considering the vulnerability probability of each node, this paper took the most likely edge and the index of the corresponding node as the predicted value $\hat{y}=argmax_{i\in \left|V\right|}\left(p_{i}\right)$ and calculated the cross-entropy loss compared with the true value.

To facilitate the model to quickly switch between different embedding methods to adapt to different embedding tasks, in addition to Word2vec, this method also encapsulates two other mainstream embedding methods, namely: GloVe [45] and FastText [46]. Bert [47] is also a common embedding method at present. It was proposed by Google in 2018. When using the Bert model for embedding, the token position needs to be considered. The current maximum length of position embedding is 512. However, the operation code of each smart contract is a very long sequence, the length of the operation sequence of some smart contracts can even exceed 3000. If Bert is used for code embedding, a large part of the operation sequence will be discarded and a lot of feature information may be lost.

### 3.3. Identifying Vulnerable Paths with a Single Model

Given an IG, this method uses the following three main steps to predict its vulnerabilities:

(1) Extract all paths and nodes.
(2) Embed all paths and nodes to obtain the embedding matrix.
(3) Use the embedded matrix as input data to make predictions.

To give the label of the contract corresponding to $IG_{i}$ conveniently, this method considers adding an artificial node that does not contain any semantic information at the position where the index of $IG_{i}$ is 0. This artificial node serves as a classification for the entire graph, indicating whether the graph contains vulnerabilities. If a sample has no loopholes, this method uses index 0 as the ground truth label of the contract. If there are loopholes, the number composed of the sequence number corresponding to the path is used as its label. For example, in the red route in the testing phase in Figure 1, the contract token will be recorded as 1246, and the probability that this route has a real vulnerability is preserved. Note that this is just one path for the example; in practice, most contracts have multiple red paths.

To identify vulnerabilities accurately, each single NN model should understand opcode sequences through multiple views. Learning such global and local combinations is necessary for every single NN model to successfully identify fragile contracts. Convolutional and recurrent neural networks can process sequential data efficiently. However, the ability to learn global dependencies is limited by the length of the corresponding path. Due to the computational cost, the acceptable path length is limited, so the independent path is usually processed. Such processing creates additional limitations for them. In contrast, Transformer allows learning latent patterns from a holistic perspective. Six models, such as CNN and RNN, pass information to edges with different weights and pass the initial vectorized node representation and edge list of each independent path to models such as CNN. It should be noted that, unlike the GNN, this paper uses the UCD, so two paths are considered; one is the sub-path in each graph and the second is the overall path of the UCD. For each sub-path, the output of models such as CNN and RNN will be the converted path vulnerability probability representation, denoted as $H^{l}=\left\{\left[h_{1}^{l.C},h_{2}^{l.C}\cdots h_{Ps}^{l.C}\right],\left[h_{1}^{l.R},h_{2}^{l.R}\cdots h_{Ps}^{l.R}\right],\cdots \right\}$, where Ps represents the number of sub-paths corresponding to each sample IG.

To learn the implicit dependencies of the sequence, this method takes all the nodes in the overall path of the sample as a sequence and passes it to the transformer to learn the vectorized representation of the overall path. Removing connecting edges forces the model to learn long-distance dependencies more efficiently since it no longer depends on the effects of node calls that are far apart. In this paper, all nodes are sorted by distance and frequency relationship, for example, the three opcodes MSSTORE, CALLVALUE, and JUMPI. If MSSTORE and CALLVALUE are placed before JUMPI in the source program, then, when building the input sequence, MSSTORE will be placed before all nodes belonging to JUMPI; CALLVALUE is also based on the same rules. With the help of the multi-head self-attention mechanism, the final node converted by the model represents the global vulnerability probability of the contract, denoted as $H^{g}=\left[h_{1}^{g},h_{2}^{g},\cdots ,h_{\left|V\right|}^{g}\right]$.

Then, the result vector representation from each model is fed into a multilayer perceptron, and this layer is used to assign weights to the models. For models such as CNN and RNN, the comprehensive score of each sub-path is calculated by Equation (1), $S^{l}=\left[s_{1}^{l},s_{2}^{l},\cdots ,s_{Ps}^{l}\right]\in {\mathbb{R}}^{Ps}$, and, for Transformer, the score of the overall path is calculated by Equation (2), $S^{g}\in {\mathbb{R}}^{\left|V\right|}$. During the training process, all vulnerability scores are passed into the softmax layer to obtain the vulnerability probability of all paths and calculate the cross-entropy loss. This paper noted that each model is trained independently, backpropagating and updating the weights without crossover.

(1) $$\begin{array}{l} s_{i}^{l}=w^{l.C}\times h_{i}^{l.C}+w^{l.R}\times h_{i}^{l.R}+w^{l.RC}\times h_{i}^{l.RC}+w^{l.D}\times h_{i}^{l.D}+w^{l.G}\times h_{i}^{l.G}+w^{l.BG}\times h_{i}^{l.BG};\\ 1\leq i\leq Ps \end{array}$$

(2) $$S^{g}=\frac{\sum_{j=1}^{\left|V\right|}\frac{h_{i}^{g}}{\sum_{i=1}^{\left|V\right|}h_{i}^{g}}\cdot h_{i}^{g}}{\left|V\right|};1\leq i,j\leq \left|V\right|$$

### 3.4. Integrating Results

**Figure 3:**
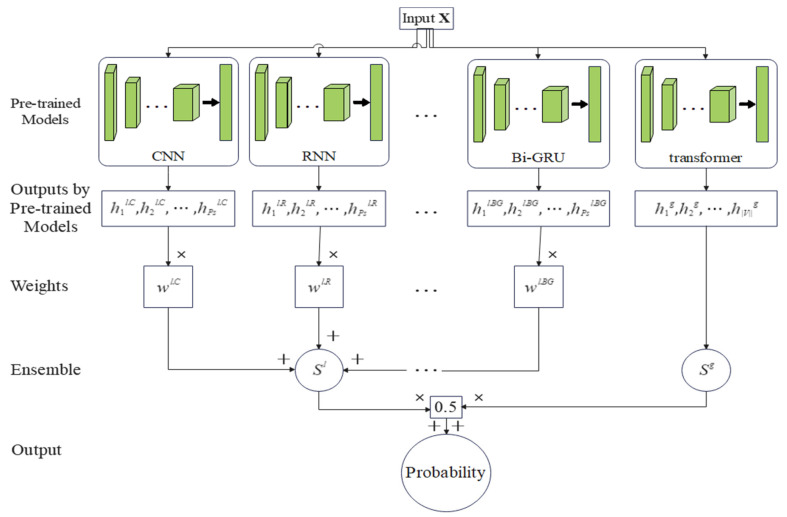
SCVDIE-Ensemble.

One of the goals of this paper is to combine the learning knowledge of multiple models, improve the accuracy and robustness of a single model, and reduce the probability of selecting a single model with poor performance. Therefore, this paper proposes an ensemble method to aggregate the prediction results of multiple models. The structure of the implemented SCVDIE-Ensemble in this study is shown in Figure 3. SCVDIE-Ensemble integrates seven mainstream classification models: CNN, RNN, RCNN, DNN, GRU, Bi-GRU, and Transformer. Using different sub-classifiers, vulnerability patterns can be learned from different aspects. In the inference phase, the user can input the vectorized data into the pretrained model to obtain the recognition result. Specifically, this paper inputs the vectorized code graphs into these seven trained models, and then they output the transformed path representations $H^{l}$ and $H^{g}$, then calculate the vulnerability scores $S^{l}$ and $S^{g}$, where $S^{l}$ is calculated by Equation (3).

(3) $$S^{l}=\frac{\sum_{j=1}^{Ps}\frac{s_{i}^{l}}{\sum_{i=1}^{Ps}s_{i}^{l}}\cdot s_{i}^{l}}{Ps};1\leq i,j\leq Ps$$

Finally, this paper summarized the prediction results through the weighted average method and calculates the sample contract vulnerability score $S^{en}$ $S^{en}=0.5\times S^{l}+0.5\times S^{g}$. If it exceeds the threshold (0.5), the contract corresponding to the graph will be considered vulnerable.

Integrating multiple methods is technically straightforward, but it is not equivalent to simple addition, which works quite well in practice [48]. In Section 5, this paper demonstrates the effectiveness of SCVDIE in reducing the size of training data and empirically demonstrates that different models can indeed learn different aspects of fragile patterns, as intuitively expected.

## 4. Study Design

### 4.1. Dataset

**Figure 4:**
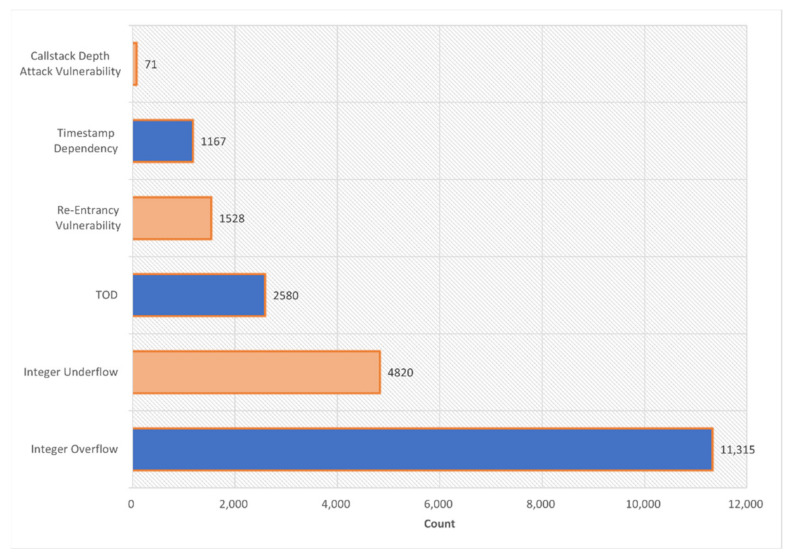
Distribution of each type of vulnerability as a percentage of the overall dataset.

**Figure 5:**
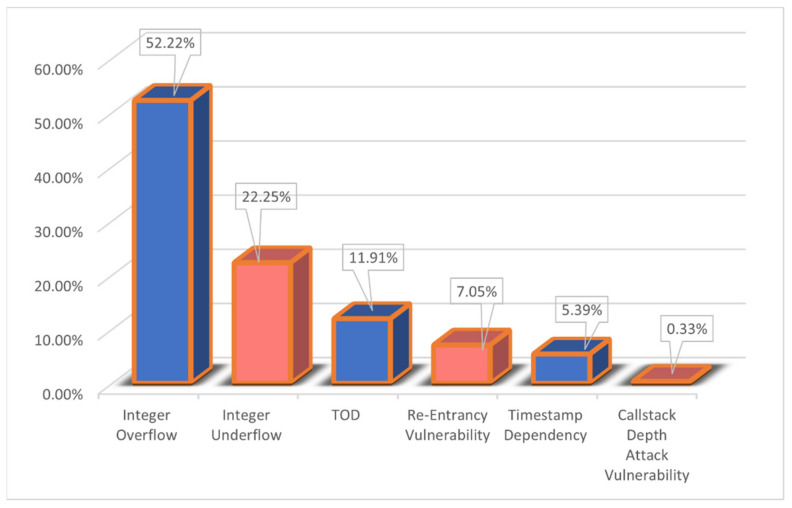
The final number of vulnerability contracts was collected.

**Figure 6:**
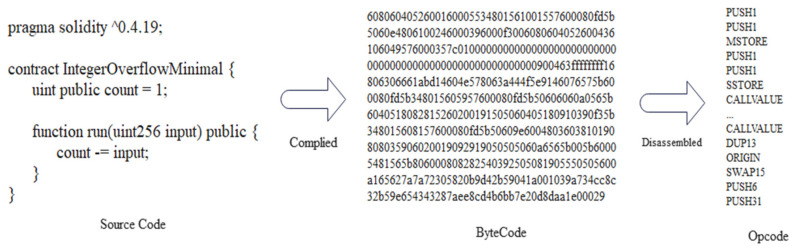
Opcode extraction process.

If given a dataset is small in size, the usual same-dataset train-test scheme would likely cause the models to overfit. So, this paper spent more than two weeks collecting more than 6000 vulnerable sol files from DEDAUB, which were written by different versions of the Solidity programing language, and, finally, 21,667 smart contracts were collected, as shown in Table 2. The dataset in this paper uses seven different types of vulnerabilities, Integer Underflow, Integer Overflow, Parity Multisig Bug 2, Callstack Depth Attack vulnerability, Transaction-Ordering Dependence (TOD), Timestamp Dependency, and Re-Entrancy vulnerability, scanned using a static parser and marked with a binary value, with 1 indicating a vulnerability and 0 indicating no vulnerability. To show the baseline dataset in more detail, we use Figure 4 to show the overall distribution of vulnerabilities on the baseline dataset. To minimize the problem of model performance degradation due to inconsistent distribution patterns in the training, validation, and test sets, the three benchmark datasets were manually made consistent in terms of the percentage of vulnerable contracts. Figure 5 shows how the final number of contracts obtained on the three benchmark datasets. In addition, the static scanner was unsuccessful in flagging Parity Multisig Bug 2 and, therefore, it is not listed. On the other hand, this paper uses the Solidity compiler to extract the opcodes from the source files. Figure 6 illustrates the process of extracting the opcode from the smart contract source code in this paper. The three text areas from left to right represent the source code, bytecode, and opcode, respectively. The leftmost text indicates the source code of the smart contract, which is written in Solidity, a high-level programing language. The middle string represents the byte code of the contract and consists of a set of hexadecimal digits. When processing smart contracts, the source code is first compiled into bytecode and then into opcodes, the most primitive data for this model. Smart contracts are stored in bytecode form on Ethereum main net but are usually published in source code form; using the opcode sequence as the analysis data makes our analyzer more flexible for use in a real-world environment [31]. Since the original opcode of each smart contract is a particularly long sequence, it would be difficult and inefficient for the model to extract features if it is fed directly into the embedding model. Therefore, this paper classifies opcodes by function, as shown in Table 3 (the classification is based on [12], but the trade-offs of opcodes are very different), and then removes all opcodes of Stack type and removes some opcodes of Compare type, such as LT, GT, etc.

Finally, the dataset constructed in this paper contains 46 different opcodes for code embedding. To confirm the validity of the IG, the original operation sequence is used as the direct embedded data in this paper as the original data for the control experiment.

### 4.2. Model Pretraining

#### 4.2.1. Dataset Split

This work first fully trained the model on 11,667 contracts in the dataset with a relatively large learning rate, and then fine-tuned the pretrained model on 10,000 contracts with a small learning rate. The inspiration for this design is that a large amount of pretraining data can grasp a part of vulnerability patterns in advance, so this paper lets the model learn this part with enough samples first, and then learn other (more complex) parts. In Section 4, this work demonstrated experimentally that the model successfully utilizes this “pre-train + fine-tune” model to transfer knowledge learned from pretraining data to unseen real-world scenarios.

**Figure 7:**
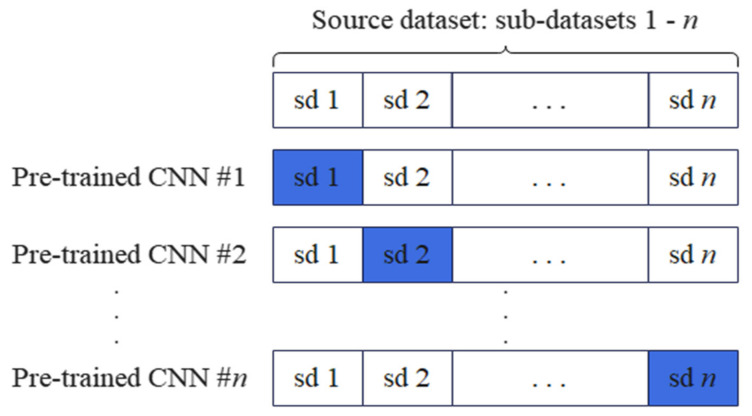
Illustration of splitting the source dataset into n-fold for pretraining n NN models.

Since n-fold CV can obtain a more reasonable and accurate evaluation of the model, especially when the dataset is small, the source dataset is first divided into n mutually exclusive subsets in the pretraining. In each experiment, the *i*th $\left(1\leq i\leq n\right)$ subset was selected as the test set (indicated by the blue block in Figure 7), while the remaining n−1 subsets were used to pretrain an NN. Therefore, an NN model was pretrained using the source dataset and tested in one experiment. Figure 7 illustrates the process of partitioning the source dataset into mutually exclusive subsets for pretraining an NN model using a CNN as an example.

#### 4.2.2. Training Algorithm

An NN model contains an unknown set of parameters, such as the weights and biases, which need to be determined during the training process. To correctly identify these parameters, a loss function CF is defined to measure the difference (or generalization error) between the model prediction and the associated truth. The parameters θ (weights ω, and deviations b) are updated utilizing SGD with momentum method to minimize the generalization error by taking small steps in the direction of the negative gradient of the loss function. This process is repeated several times, with each iteration performed on a small number of training samples, until the generalization error is close to zero. The loss function $CF\left(\theta \right)$ is defined as:

(4) $$CF_{R}\left(\theta \right)=CF\left(\theta \right)+ƛ\psi \left(\omega \right)=\frac{\sum_{i=1}^{k}{\left(\eta_{\theta }\left(x^{\left(i\right)}\right)-y^{\left(i\right)}\right)}^{2}}{2k}+\frac{ƛ}{2}\omega^{T}\omega$$

where $CF_{R}\left(\theta \right)$ denotes the loss function, $ƛ\psi \left(\omega \right)$ denotes the regularization term, ƛ denotes the $L_{2}$ regularization factor, which weighs the relative contribution of the penalty term $\psi \left(\omega \right)$, $\eta_{\theta }\left(x\right)$ denotes the hypothesis function, k denotes the number of samples used in each iteration, $x^{\left(i\right)}$ denotes the *i*th input sample matrix, and $y^{\left(i\right)}$ denotes the corresponding target value.

The hypothesis function $\eta_{\theta }\left(x\right)$ can be expressed as:

(5) $$\eta_{\theta }\left(x\right)=b_{0}x_{0}+\sum_{j=1}^{n}w_{j}x_{j}$$

where $w_{n}$ and $x_{n}$ denote the nth unknown parameter and its corresponding input variable, respectively. Note that $b_{0}$ is a bias and $x_{0}=1$.

The parameter θ can be updated iteratively in the following way:

(6) $$\vartheta =\frac{1}{2q}\sum_{i=1}^{q}{\left(\eta_{\theta }\left(x_{j}^{\left(i\right)}\right)-y_{j}^{\left(i\right)}\right)}^{2}$$

(7) $$\theta_{j+1}=\theta_{j}-\alpha \vartheta +\gamma \left(\theta_{j}-\theta_{j-1}\right)-ƛ\alpha \theta_{j}$$

where ϑ denotes the exact gradient estimator for sampling the mini-batch of q samples, and $x_{j}^{\left(i\right)}$ denotes the ith input matrix of the mini-batch in the jth iteration. $y_{j}^{\left(i\right)}$ denotes the corresponding target value, α denotes the update step (or initial learning rate), γ denotes the momentum, which determines the gradient effect of the previous iteration on the current iteration, and $\theta_{j}$ denotes the parameter estimate of the jth iteration.

The pseudo-code for the model training process is shown in Algorithm 1.
**Algorithm 1:** Model training algorithm.1: Read Data dt
2: Pre-train the model3: for model in CNN, RNN, …, Transformer do4:     for *i* in 1, 2, 3, 4, 5 do5:             1. Batch and Shuffle dt by Mini-batch size to Generate D
6:             2. Randomly divide D as the training set $D_{t}$, validation set $D_{v}$, test set $D_{tt}$
7:             Initialize ω and b randomly by the Gaussian distribution8:             Reading parameter configuration information9:             for epoch in 1 to 20 do10:                 3. $va=matmul\left(x,\omega \right)$ # calculate the value before activation11:                 4. $ypre=softmax\left(va\right)$ # the value after activation12:                 5. $CF\left(\theta \right)$ = Equation (4) # compute loss13:                 6. $grd=sgdwithmomentum\left(CF\left(\theta \right),\theta \right)$
14:                 7. $\eta_{\theta }\left(x\right)$ = Equation (5) # fit the regression curve15:                 8. $\theta_{j+1}$ = Equation (7) # update model parameters16:           End17:     End18: End19: Fine-Tune.

The time complexity determines the efficiency of the model. If the time complexity is too high, training a proven model takes a lot of time and not only does it not make predictions fast enough, it also makes it challenging to fine-tune the parameters. The spatial complexity is determined by the number of parameters of the model. Because there is a dimensional catastrophe, the more parameters the model has, the more data are required to train the model. It is, therefore, necessary to analyze the complexity of the SCVDIE. For a CNN, the time complexity is expressed as $Time∼O\left(\sum_{l=1}^{De}M_{l}^{2}\cdot K_{l}^{2}\cdot C_{l-1}\cdot C_{l}\right)$, $Space∼O\left(\sum_{l=1}^{De}K_{l}^{2}\cdot C_{l-1}\cdot C_{l}+\sum_{l=1}^{De}M^{2}\cdot C_{l}\right)$ denotes spatial complexity, De indicates network depth, l denotes the l-th convolutional layer, $C_{l}$ denotes the number of output channels of the l-th convolutional layer, i.e., the number of convolutional kernels, the number of input channels, $C_{in}$ is the number of output channels of the previous convolutional layer, K denotes the convolutional kernel size of the l-th convolutional layer, and $M^{2}$ denotes the spatial dimensions of a feature. For RNN and GRU, the overall computational complexity is expressed as $O\left(n\cdot m+n^{2}+n\right)$; here, n is the hidden size and m is the input size. Note that the actual computational complexity of GRU is less than this value, and the two are unified in this paper to reduce the complexity of the representation. For DNNs, the time complexity of the matrix multiplication is $O\left(n^{3}\right)$; by having the same number of neurons per layer, then there are $O\left(n\cdot n^{3}\right)=O\left(n^{4}\right)$. For transformers that use self-attention, the complexity of the similarity calculation is $O\left(n\cdot d\cdot n\right)=O\left(n^{2}d\right)$, the complexity of the softmax calculation is $O\left(n\right)$, and the weighted sum is $O\left(n^{2}d\right)$.

SCVDIE provides a profile with training and testing parameters in dictionary format, providing detailed options and help for optimizing model performance. Users can pretrain the model directly using the default settings or customize the training process by fine-tuning the parameter settings and hyperparameters. SCVDIE also provides one-click execution scripts that allow users to invoke different modules of SCVDIE to accomplish various tasks by specifying model parameters in the configuration file. For example, the user can specify the value of the model parameter, which can be train_word2vec, train_Glove, train_FastText, train_classifier, interactive_predict, save_model, and test, to allow SCVDIE to train the embedded model, train the classifier, test the classification, save the model for the tf-severing interface, and single or batch test, respectively. In addition, SCVDIE also supports users to select different code embedding methods and classifiers through profiles.

### 4.3. Experimental Configuration

Another goal of the proposed EL approach in this paper is to reduce the expected generalization error defined by Equation (4). To achieve this goal, seven different NN models are pretrained in this paper. Table 4 lists the values of several important parameters used to pretrain the NN model. The pretraining was performed with an SGD with momentum, and the mini-batch size was set to 128. The initial learning rate of all layers is set to $10^{-3}$, and then the learning rate is adjusted by the warmup strategy [49] to obtain better convergence, i.e., the learning rate is reduced by a factor of 2 every five training epochs. The weights of each layer are randomly initialized according to the Gaussian distribution. The Gaussian distribution has a mean of 0 and a variance of 1. The bias value of each layer is initialized to 0. To prevent model overfitting, the early stopping method is used to automatically terminate the model. Specifically, if the root mean square error (RMSE) of the validation set is greater than or equal to the minimum RMSE of the previous validation set in five consecutive epochs, then the network training is stopped.

In the fine-tuning phase, the hyperparameters (i.e., learning rate and epochs) are chosen based on the performance of the target task on the validation set, and the learning rate is set to $10^{-5}$. The learning rate is chosen to maximize the performance of the target task with a reasonable number of epochs; epochs are set to 60. Note that the epochs are carefully chosen rather than set as large or small as possible, and the choice is based on the fact that the validation loss of all models stops decreasing in five consecutive epochs. The weights and biases of each layer are set to the values transferred from the corresponding pretrained model.

In addition, all models in this paper are based on TensorFlow v2.3.0, CUDA v10.1.243, and trained with an 11GB Nvidia GeForce RTX 1080Ti GPU. It takes 15 min to train Word2vec. The model takes 8.2 h to train on the pretrained data and 15 h on the fine-tuned data.

### 4.4. Performance Evaluation

**Figure 8:**
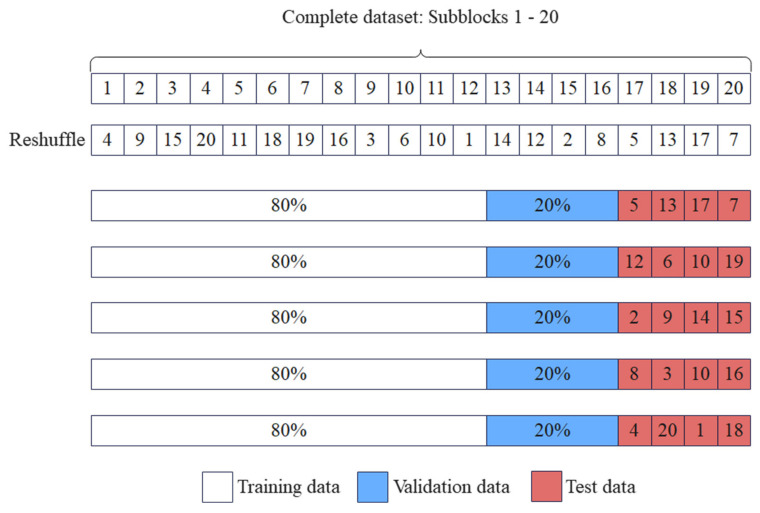
The fivefold CV process. In addition to the test set, the training set in each CV experiment consists of 80% of the dataset, while the remaining 20% is used to construct the validation set.

The performance of the proposed SCVDIE-Ensemble model is evaluated by the method shown in Figure 8, i.e., the accuracy of the proposed SCVDIE-Ensemble model is evaluated using a fivefold CV. This paper first divided the complete dataset into 24 mutually exclusive sub-blocks. Subsequently, five CV experiments were performed. In each CV experiment, these sub-blocks were randomly disrupted, and then four sub-blocks were randomly selected for testing, while the remaining sub-blocks were divided into a training set (16 sub-blocks, 80%) and a validation set (4 sub-blocks, 20%). In the kth CV experiment, the SCVDIE-Ensemble model was used to estimate the ability of the test set (i.e., from the corresponding four sub-blocks) samples. After conducting all five CV experiments, the overall performance of the SCVDIE-Ensemble was evaluated by fuzzy matrix and test errors $R_{RMS}^{t}$, where the overall test error $R_{RMS}^{t}$ is obtained by calculating the mean of the test error $R_{RMS}^{k}$ for five individual CV experiments, which reflects the performance of the proposed SCVDIE-Ensemble model through Equation (9).

(8) $$R_{RMS}^{k}=\sqrt{\frac{\sum_{i=1}^{N_{te.k}}{\left(y^{k}(\nu_{i}^{k})-{\hat{y}}^{k}(\nu_{i}^{k})\right)}^{2}}{N_{te.k}}}$$

(9) $$R_{RMS}^{t}=\sqrt{\frac{\sum_{k=1}^{5}\sum_{i=1}^{N_{te.k}}{\left(y^{k}(\nu_{i}^{k})-{\hat{y}}^{k}(\nu_{i}^{k})\right)}^{2}}{\sum_{k=1}^{5}N_{te.k}}}$$

where $N_{te.k}$ denotes the number of contracts used for testing in the kth CV experiment. $\nu_{i}^{k}$ denotes the input feature vector of the *i*th sample in the kth CV experiment, and $y^{k}(\nu_{i}^{k})$ and ${\hat{y}}^{k}(\nu_{i}^{k})$ denote the true label and predicted label of the *i*th sample in the kth experiment, respectively.

### 4.5. Baselines

One of the main objectives of the proposed deep-learning-based vulnerability detection tool in this paper is to overcome the drawbacks of rule-based static analyzers. This paper uses three static tools, Oyente [21], Mythril [22], and Securify [26], as baselines to demonstrate the improvements brought by SCVDIE-Ensemble. Moreover, this paper compared the different models in Figure 1 by replacing the model architecture. For example, SCVDIE-Ensemble is compared with pure CNN and pure transformer models. In the experiments, the input consists of preprocessed graph nodes, since this paper uses node embedding instead of token embedding.

## 5. Results and Discussion

This paper evaluated SCVDIE through the following research questions:

RQ1: Vulnerability assessment and classification. Can SCVDIE detect static scan tool tags without vulnerabilities? How big is the gap with unsupervised learning without static tool labels?

RQ2: Ensemble strategy evaluation. Is the integrated model an effective method for combining global and local environments compared to existing methods? Does the dynamic change of the prediction result weights have any effect on the global result?

### 5.1. RQ1: Vulnerability Assessment and Classification

First, this paper tests the ability of SCVDIE-Ensemble to identify vulnerable contracts in a realistic environment. In a real-world environment, there is no way to know in advance if a contract is vulnerable. This simulates a typical static analysis environment in which the static analyzer aims to find as many contractual vulnerabilities as possible. The experiments in this paper can be considered as vulnerability assessment and classification, where SCVDIE classifies a smart contract as vulnerable/invulnerable, and then gives a determination result by combining different patterns learned from multiple models. In RQ1, this paper focuses on the comparison between SCVDIE-Ensemble and rule-based static analysis tools. This discussion is given priority in this paper because one of the goals of this paper is to improve the overall performance of static analysis tools using a data-driven approach to further ease the debugging efforts of developers. To comprehensively evaluate the effectiveness of the method in this paper, SCVDIE is compared with a static analyzer and a variant of SCVDIE. They are SCVDIE-Ensemble, SCVDIE-CNN, SCVDIE-RNN, SCVDIE-RCNN, SCVDIE-DNN, SCVDIE-GRU, SCVDIE-Bi-GRU, and SCVDIE-Transformer. As mentioned in Section 3.1, to incorporate nonvulnerable contracts into SCVDIE, this paper adds an artificial node to each virtual node with index 0 without any syntactic and semantic information. If the sample is vulnerability-free, then $y^{k}(\nu_{i}^{k})$ will be marked as 0, which is the index of the artificial node; otherwise, $y^{k}(\nu_{i}^{k})$ will be a value consisting of the indexes of the nodes contained in the vulnerability path.

Table 5 and Table 6 show the comparative results of RMSE, accuracy, precision, recall, F1, and prediction accuracy of different models on the combination of fragile and nonfragile contracts, respectively. In view of these results, three significant observations can be made and are listed as follows.

**Figure 9:**
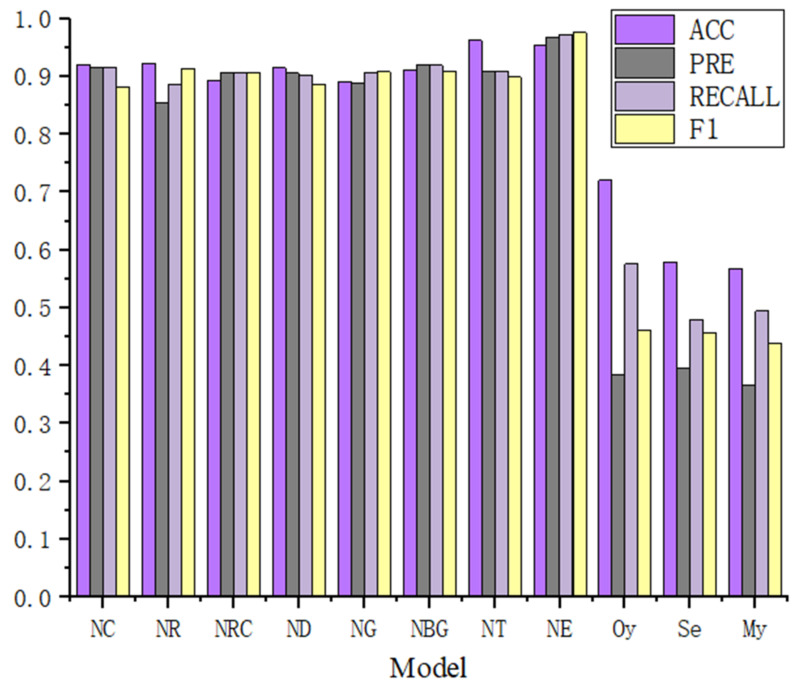
Model performance comparison.

First, based on these results, the performance of the SCVDIE-ENSEMBLE was better than that of the SCVDIE-CNN, SCVDIE-RNN, SCVDIE-RCNN, SCVDIE-DNN, SCVDIE-GRU, SCVDIE-Bi-GRU, and SCVDIE-Transformer methods, as shown in Figure 9. Combining the RMSEs and fuzzy matrix results, SCVDIE-Ensemble is also able to make more accurate vulnerability predictions in a single CV. Although the RMSEs of SCVDIE-Ensemble do not remain the lowest in every CV, the combined RMSE of SCVDIE-ENSEMBLE outperforms other methods, which means that SCVDIE-ENSEMBLE can still maintain a low error in obtaining high accuracy prediction results.

Second, the combined RMSE of SCVDIE-ENSEMBLE is 1.419%, which indicates that the proposed method can provide more accurate vulnerability information, even if the training set is relatively small. When compared with other variants of SCVDIE, for example, SCVDIE-Transformer (3.395%) and SCVDIE-CNN (5.039%), it can be concluded that combining different knowledge learned by different neural networks helps to improve the vulnerability prediction generalization of any single model.

Third, SCVDIE significantly outperformed static analysis tools, reducing the false-positive and false-negative rates dramatically. The best performing SCVDIE-ENSEMBLE achieved 97.57% of the evaluated F1 scores, while the rule-based static analysis tool could only achieve 46.1% accuracy at most. In the contract-level vulnerability detection setup, the SCVDIE variant has significantly higher precision, recall, and F1 than all baseline static analysis tools. These results objectively validate the fact that SCVDIE helps to alleviate developers’ concerns about the FP/FN problem of rule-based static analyzers.

Overall, these results show the effectiveness of the integrated approach when combined with the seven-class model. However, this paper also observed that the single Transformer model is more accurate than the integrated model in terms of classification accuracy, although this value is within 1%. This is because the global nature of the Transformer model allows it to perform better on more general vulnerability detection tasks, but this advantage is slightly diminished when combined with models such as CNN.

### 5.2. RQ2: Aggregate Strategy Evaluation

This paper had demonstrated the effectiveness of the integrated model in global and local vulnerability assessment. The model is more effective globally compared to a single model in terms of a comprehensive understanding of global and local factors.

The integration strategy of SCVDIE outperforms existing models that aggregate global and local contexts. As shown in Table 7, the integrated approach shows a more powerful learning capability compared to existing models. Compared with the existing models studied in this paper, its learning capability is more powerful. SCVDIE-ENSEMBLE outperforms other single policies in both the vulnerability-only and mixed settings. Specifically, in the real world, SCVDIE-ENSEMBLE illustrates the versatility of the integration approach to understand complex real-world vulnerability patterns. This result empirically suggests that integrating models is a more direct and effective way to combine global and local knowledge than stacking different models, since stacked models still share learned knowledge during training, which may prevent them from learning a more diverse set of vulnerability patterns.

### 5.3. Effect of the Training Dataset Size

**Figure 10:**
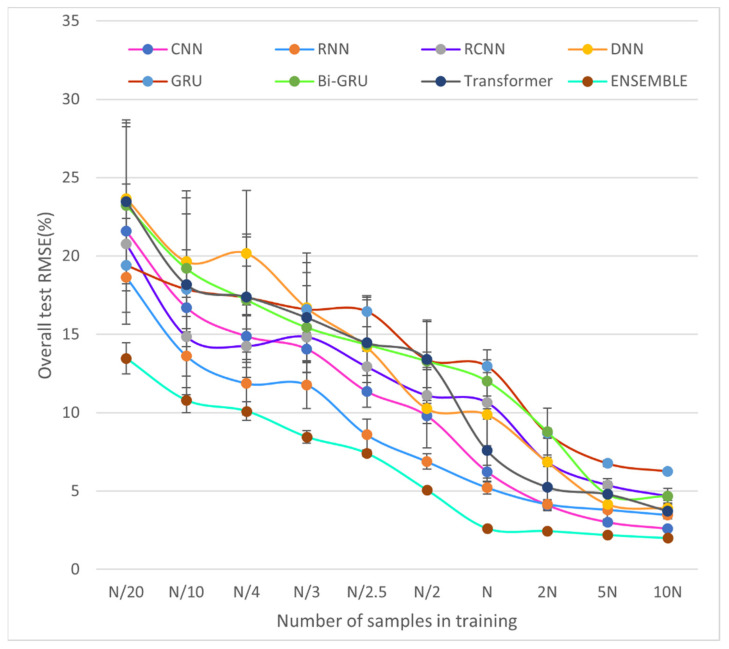
Comparison of model errors under different size training datasets.

The number of smart contracts used in this paper is only the tip of the iceberg of many contracts, and the relative reliability of the experimental results cannot be guaranteed without more datasets. To thoroughly investigate the impact of using a relatively small dataset on the performance of the proposed SCVDIE-ENSEMBLE model, we dynamically change the number of samples in the original training dataset and construct 10 sub-training datasets of different sizes. The maximum number of samples in each training dataset is called $n_{max}$, and the range of values is N/20 to 10N, $n_{max}\in \left\{N/20,N/10,N/4,N/3,N/2.5,N/2,N,2N,5N,10N\right\}$. When $n_{max}=N$, there is no need to make any adjustments to the original dataset used. To validate the generalization of the model, each of these 10 training sets used the same test dataset during the testing phase. Moreover, the SCVDIE-ENSEMBLE model was compared with variants of SCVDIE trained with datasets of different sizes. As is known, NNs trained from the small-scale training set may suffer from overfitting problems. Especially when the number of samples in the training set does not exceed N/2, i.e., $n_{max}\leq N/2$, this phenomenon is easier to detect. Figure 10 shows the performance changes of different models with the size of the training set, from which the following conclusions can be drawn.

First, when comparing different independent models and ensemble models, it can be found that the performance of the ensemble model is greatly improved compared to that of the independent model because different deep neural networks can recognize different patterns of features. The performance improvement of SCVDIE-ENSEMBLE becomes obvious as the training data set decreases, which indicates that EL is a powerful tool to improve vulnerability prediction precision, which further supports the inference made earlier in this paper. The SCVDIE-ENSEMBLE converged near $n_{max}=N$; the number is more than five times that required for a standalone NN to achieve a satisfactory horizon of performance. For example, the overall average RMSE of SCVDIE-DNN = 6.854% at $n_{max}=5N$. With the number of training samples increasing, the performance boost from SCVDIE-ENSEMBLE diminishes gradually. This is because, when there are enough training data samples, a single model can also grasp a relatively more comprehensive feature pattern. In other words, each independent model can achieve similar performance at the expense of a larger training dataset. In the case of $n_{max}=N$, the RMSE metric of SCVDIE-ENSEMBLE is 2.456%, while each independent model requires a training dataset 5 to 10 times larger than SCVDIE-ENSEMBLE to reduce the error to this level. However, this requires a greater investment of labor and time costs to obtain larger datasets. Thereby, in the case of limited time and samples, using EL instead of training individual NNs is a better method to improve the accuracy of smart contract vulnerability prediction.

Second, SCVDIE-ENSEMBLE has a shorter error range compared to the standalone NN model. This suggests that more confident vulnerability prediction can be obtained by the combination of NN and EL than by not using EL. The SCVDIE-ENSEMBLE model consists of seven different NN models, each of which can be responsible for a different focus, and more patterns can be learned under the same conditions, which is also consistent with the first conclusion.

Finally, as mentioned at the beginning of this subsection, training a complex deep learning model with a small amount of data can lead to overfitting. Although the proposed SCVDIE-ENSEMBLE model has a complex structure consisting of seven individual NN models and the training dataset used in this study is relatively small (i.e., $n_{max}=N$), the model is not expected to be overfitted in this paper. The SCVDIE-ENSEMBLE was tested by comparing the overall test RMSE achieved when trained on a progressively larger dataset (i.e., $n_{max}=2N,5N,10N$) with the RMSE achieved when trained using the original dataset (i.e., $n_{max}=N$). The overall RMSEs converge almost to a horizontal asymptote when the size of the training dataset increases, which suggests that the overall test RMSEs do not decrease significantly if more training samples are provided. Therefore, the proposed SCVDIE-ENSEMBLE model does not appear to be overfitted.

The above conclusions are a preliminary analysis of the experimental results, mainly for the comparison of SCVDIE-ENSEMBLE with other models, but still lacking in the analysis of other single models. We will discuss the results more fully and in greater depth below. The first to say is that the CNN and RNN models, which are closer to the performance of the integrated model, are more similar. In Figure 10, it can be seen that, when the dataset is small, the loss value of CNN is greater than RNN, but, when the sample of the dataset increases, the advantage of RNN disappears and, instead, CNN achieves good results. This indicates that the CNN requires a higher amount of data than the RNN in this task. In addition, both CNNs showed better recognition results when the number of data samples increased rapidly, which can be interpreted as a higher performance of CNNs than RNNs in this task when there are enough samples. Secondly, this paper also identifies an interesting phenomenon. In NLP, Transformer has achieved significant performance gains over other models, yet its performance in this task is mediocre. This paper argues that this may be because, although the Transformer can handle longer sequences of operations and retain more information than other single models, and this paper also preprocesses the sequences to reduce their length, there are still a small number of contract sequences that are too long, and these overly long sequences lead to a lot of data noise that affects the Transformer’s performance. In contrast, the rest of the single model deals with data that are a sequence corresponding to each sub-path, which is relatively much smaller in length and more conducive to adequate feature extraction. Another object that deserves to be discussed is the GRU model, due to its relatively worst performance. GRU and Bi-GRU as optimization models for RNNs can solve the RNN gradient vanishing problem, but they also bring another potential problem: a large number of parameters, where GRU has three times more parameters than Navie RNN, which seriously increases the model spatial complexity. Therefore, this explains the superiority of the RNN model when dealing with relatively short sequences of sub-path operations.

## 6. Further Discussions

The specialty of this paper’s approach in dealing with smart contracts. The opcode of a smart contract is a sequence of specific characters, containing many nonlinear call relations, similar to the assembly language of a traditional program. In contrast to existing methods of extracting contract sequences using source code, this paper combines both the sequence features of individual contracts and the overall features. This has the obvious advantage of not only extracting features from a single linear sequence of operands, but also of segmenting them to find potential features in multiple dimensions, which greatly reduces the length of a single input sequence and effectively reduces the dimensionality of the data embedding. To this end, we have also built a unique opcode-based IG structure diagram for the first time, which better represents the relationship between the local and the whole. Ultimately, the superior performance of the proposed method in this paper was demonstrated in a quantitative manner using extensive experiments. Both the mean F1 score and the mean prediction accuracy achieved optimal values of 97.57% and 97.42%, respectively, and achieved lower relative RMSEs on different size datasets.

Findings. EL has been successful in a variety of areas, yet little has been reported on blockchain and IoT security. To this end, this paper designs a novel EL model and conducts multiple sets of comparative experiments using real-world smart contracts. The results confirm that EL can produce better results with fewer data to train. This performance improvement over popular methods, such as symbol-based execution, is easy to explain. This is because methods based on symbolic execution face significant challenges, such as path explosion, which limits the performance of these popular methods. This performance improvement over a single-model-based approach can be attributed to the fact that different classification models can specialize in different parts of the dataset. In other words, different models focus on different vulnerability features, which improves the performance of SCVDIE while paving the way for less dependence on the dataset for SCVDIE. This is because every single NN focuses on a different vulnerability feature that the integrated model can be used to learn more features when the dataset is limited, thus increasing the utilization of the dataset.

Limitations. Despite these new findings, the following limitations of this work remain. For example, while SCVDIE can reduce the amount of data required by the model, and thus reduce the human and time costs required to collect the data, this implementation is indirect and does not directly address the resources spent on collecting a certain amount of vulnerability data. In addition, this paper uses binary numbers to indicate whether a contract contains vulnerabilities and, in the future, will explore how to make the classification results more intelligent. For example, exploring how to output separate results for different vulnerabilities.

## 7. Conclusions and Future Work

In this study, we proposed innovatively an EL-based approach called Ensemble Learning Based Smart Contract Vulnerability Prediction (SCVDIE-ENSEMBLE) to predict vulnerabilities in Ethereum smart contracts. The proposed SCVDIE-ENSEMBLE method incorporated multiple NNs, which are CNN, RNN, RCNN, DNN, GRU, Bi-GRU, and Transformer. Each NN has its unique role to play, which allows SCVDIE-ENSEMBLE to increase the efficiency of data utilization while having a more accurate and robust performance on unseen data. We have also investigated a novel approach to data classification that underpins a wide range of experiments.

We have proven the performance of SCVDIE-ENSEMBLE with numerous experiments. First, a fivefold CV experiment was conducted on the benchmark dataset for the seven single models and the overall model integrated by SCVDIE-ENSEMBLE. Quantitative experimental results demonstrate that SCVDIE-ENSEMBLE has a smaller error in classification results. Next, the performance of these eight models and three popular methods are compared. The average prediction accuracy of SCVDIE-ENSEMBLE was experimentally demonstrated to be better than other methods. The performance of the SCVDIE-ENSEMBLE integration strategy is then further demonstrated by comparison with the general approach. Finally, the contribution of SCVDIE-ENSEMBLE to reducing the model’s reliance on large datasets, and hence the cost of data collection, is demonstrated in multiple dimensions by varying the size of the dataset.

We believe this work is an important step toward alleviating the challenges of data collection and IoT security. For future work, since the SCVDIE-ENSEMBLE model relies on existing learnable features, as with other NNs, we will focus on breaking this limitation by combining the underlying NN model with other deep learning methods, such as transfer learning that can be extended to similar domains.

## Figures and Tables

**Table 1:** Symbols and corresponding descriptions.

Symbol	Description	Symbol	Description
IG	IG of a contractual composition, not specific.	$d_{h}$	The hidden dimension.
V	The set of nodes of IG.	W	The linear layer weight.
E	The set of edges of IG.	b	The linear layer bias.
D	Dataset.	P	Probability of results.
$IG_{i}$	The *i*-th contract constitutes the IG.	$\hat{y}$	Predictive labeling of the sample.
$y_{i}$	Data labels corresponding to $IG_{i}$.	$p_{i}$	The probability of the *i*-th path.
N	The original number of samples.	Ps	the number of sub-Paths
$\left|V\right|$	The number of nodes of $IG_{i}$.	$h_{i}^{l.C}$	Vector of the *i*-th sub-path on CNN.
F	The node embedding method.	$h_{i}^{l.R}$	Vector of the *i*-th sub-path on RNN.
$v_{i}$	The *i*-th node of IG.	$H^{l}$	Integral vector of sub-paths.
$\beta_{i}$	Embedded vectors corresponding to $v_{i}$.	$h_{j}^{g}$	Vector of the *j*-th sub-path on Transformer.
ℝ	Value Domain.	$H^{g}$	Vector of the overall path on Transformer
d	Embedding dimensions.	$S^{l}$	The combined score of sub-paths.
B	The set of $\beta_{i}$.	$s_{i}^{l}$	The score of the *i*-th sub-path.
y	Data labels corresponding to IG.	$w^{l.C}$	Sub-paths result in weights on CNN.
$B^{\prime }$	Overall node information corresponding to IG.	$S^{g}$	Overall path composite score.
$\beta^{\prime }$	the node semantic embedding.	$S^{en}$	Final prediction results for the sample.

**Table 2:** Distribution of raw data of smart contracts.

Version	Sol Files	Number	Version	Sol Files	Number
0.4.0	92	238	0.4.11	737	4375
0.4.1	7	14	0.4.12	44	291
0.4.2	110	438	0.4.13	404	2348
0.4.3	3	30	0.4.14	31	130
0.4.4	634	2028	0.4.15	270	1652
0.4.5	4	4	0.4.16	650	1913
0.4.6	97	182	0.4.17	169	796
0.4.7	18	34	0.4.18	1393	7910
0.4.8	255	1425	0.4.19	423	2845
0.4.9	83	196	0.4.20	1097	5143
0.4.10	90	276	0.4.21	188	919

**Table 3:** Operation code category division.

Type	Instructions
Calldata&Codedata	callcode, calldatacopy, callvalue, calldataload, calldatasize, codecopy, codesize, extcodecopy
Jump&Stop	stop, jump, junpi, pc, returndatacopy, return, returndatasize, revert, invalid, selfdestruct
Memory	mload, mstore, msize, sstore, call, create, delegatecall, staticcall
Compute	Add (x, y), addmod (x, y, m), div (x, y), exp (x, y), mod (x, y), mul (x, y), mulmod (x, y), sdiv (x, y), signextend (i, x), smod (x, y, m)
Compare	Eq (x, y), iszero (x), gt (x, y), lt (x, y), sgt (x, y), slt (x, y)
Block	gasprice, gaslimit, difficulty, number, timestamp, coinbase, blockhash (b), keccak256
Transaction	caller, gas, origin, address, balance
Bitoperation	And (x, y), byte (n, x), not (x), or (x, y), shl (x, y), shr (x, y), sar (x, y), xor (x, y)
Stack	dup, log, pop, push, swap

**Table 4:** List of parameter values used in the NN pretraining and SCVDIE-Ensemble.

Parameter	Pretraining	Fine Tune
Initial learning rate, learning_rate	$10^{-3}$	$10^{-5}$
Mini-batch size	128	64
Momentum, ρ	0.9	0.9
$L_{2}$ Regularization, ƛ	0.0001	0.0003
epochs	20	60
Embed Dimension	180	180
Number of convolution kernels	128	128
Dropout Rate	0.3	0.3
Hidden Dimension $d_{h}$	2048	2048

**Table 5:** Error assessment (%).

Model	CV 1	CV 2	CV 3	CV 4	CV 5	Overall
SCVDIE-Ensemble	1.332	1.160	0.948	1.137	2.205	1.419
SCVDIE-CNN	3.132	2.083	8.228	2.045	3.560	5.039
SCVDIE-RNN	9.501	5.495	1.399	6.854	2.086	4.768
SCVDIE-RCNN	2.265	5.170	1.247	5.815	1.104	3.505
SCVDIE-DNN	3.977	1.693	4.668	4.338	6.793	3.860
SCVDIE-GRU	4.703	1.389	12.271	1.105	1.445	4.117
SCVDIE-Bi-GRU	2.117	2.523	8.187	3.355	6.500	3.450
SCVDIE- Transformer	1.043	1.191	3.911	2.764	4.787	3.395

**Table 6:** Performance evaluation.

Approach	Performance Indicators				
	Accuracy (avg.)	Precision (avg.)	Recall (avg.)	F1 (avg.)	Prediction Accuracy (avg.)
SCVDIE-Ensemble	95.46%	96.81%	97.26%	97.57%	97.42%
SCVDIE-CNN	92.00%	91.57%	91.50%	88.18%	90.75%
SCVDIE-RNN	92.29%	85.44%	88.68%	91.26%	88.87%
SCVDIE-RCNN	89.34%	90.66%	90.56%	90.54%	90.87%
SCVDIE-DNN	91.46%	90.53%	90.19%	88.70%	87.12%
SCVDIE-GRU	89.11%	88.81%	90.52%	90.78%	90.05%
SCVDIE-Bi-GRU	91.19%	91.88%	91.87%	90.89%	91.19%
SCVDIE-Transformer	96.20%	90.89%	90.88%	89.88%	91.00%
Oyente	72%	38.5%	57.6%	46.1%	N/A
Securify	57.9%	39.6%	48.0%	45.6%	N/A
Mythril	56.8%	36.5%	49.4%	43.9%	N/A

**Table 7:** Comparison with existing methods.

Model	F1 Score	
	SCVDIE	Common Method
SCVDIE-Ensemble	97.42%	90.51%
SCVDIE-CNN	88.18%	89.57%
SCVDIE-RNN	91.26%	81.54%
SCVDIE-RCNN	90.54%	81.06%
SCVDIE-DNN	88.70%	91.53%
SCVDIE-GRU	90.78%	89.81%
SCVDIE-Bi-GRU	90.89%	82.70%
SCVDIE-Transformer	89.88%	91.90%

## Data Availability

Self-built datasets were analyzed in this study. The source code has been made available at https://github.com/yzu-wjl/SCVDIE (accessed on 30 March 2022).

## References

[1] Lade P., Ghosh R., Srinivasan S. (2017). Manufacturing analytics and industrial internet of things. IEEE Intell. Syst..

[2] Alzubi O.A., Alzubi J.A., Shankar K., Gupta D. (2021). Blockchain and artificial intelligence enabled privacy-preserving medical data transmission in Internet of Things. Trans. Emerg. Tel. Tech..

[3] Jeon S., Lee G., Kim H., Woo S.S. SmartConDetect: Highly Accurate Smart Contract Code Vulnerability Detection Mechanism using BERT. Proceedings of the 2021 KDD Workshop on Programming Language Processing.

[4] Mehar M.I., Shier C.L., Giambattista A., Gong E., Fletcher G., Sanayhie R., Kim H.M., Laskowski M. (2019). Understanding a revolutionary and flawed grand experiment in blockchain: The DAO attack. J. Cases Inf. Technol. JCIT.

[5] Palladino S. (2017). The Parity Wallet Hack Explained. OpenZeppelin.

[6] Jiang B., Chen Y., Wang D., Ashraf I., Chan W.K. WANA: Symbolic Execution of Wasm Bytecode for Extensible Smart Contract Vulnerability Detection. Proceedings of the 2021 IEEE 21st International Conference on Software Quality, Reliability and Security (QRS).

[7] Liu Y., Xu J., Cui B. (2021). Smart Contract Vulnerability Detection Based on Symbolic Execution Technology. Communications in Computer and Information Science, Proceedings of the China Cyber Security Annual Conference, Beijing, China, 20–21 July 2021.

[8] Wang Z., Wen B., Luo Z., Liu S. (2021). MAR: A Dynamic Symbol Execution Detection Method for Smart Contract Reentry Vulnerability. Communications in Computer and Information Science, Proceedings of the International Conference on Blockchain and Trustworthy Systems, Guangzhou, China, 5–6 August 2021.

[9] Samreen N.F., Alalfi M.H. Smartscan: An approach to detect denial of service vulnerability in ethereum smart contracts. Proceedings of the 2021 IEEE/ACM 4th International Workshop on Emerging Trends in Software Engineering for Blockchain (WETSEB).

[10] Aidee N.A.N., Johar M.G.M., Alkawaz M.H., Hajamydeen A.I., Al-Tamimi M.S.H. Vulnerability Assessment on Ethereum Based Smart Contract Applications. Proceedings of the 2021 IEEE International Conference on Automatic Control & Intelligent Systems (I2CACIS).

[11] Huang J., Zhou K., Xiong A., Li D. (2022). Smart Contract Vulnerability Detection Model Based on Multi-Task Learning. Sensors.

[12] Sun Y., Gu L. (2021). Attention-based machine learning model for smart contract vulnerability detection. J. Phys. Conf. Ser..

[13] Xu Y., Hu G., You L., Cao C. (2021). A Novel Machine Learning-Based Analysis Model for Smart Contract Vulnerability. Secur. Commun. Netw..

[14] Fan Y., Shang S., Ding X. (2022). Smart Contract Vulnerability Detection Based on Dual Attention Graph Convolutional Network. Lecture Notes of the Institute for Computer Sciences, Social Informatics and Telecommunications Engineering, Proceedings of the International Conference on Collaborative Computing: Networking, Applications and Worksharing, Suzhou, China, 16–17 October 2021.

[15] Mi F., Wang Z., Zhao C., Guo J., Ahmed F., Khan L. VSCL: Automating Vulnerability Detection in Smart Contracts with Deep Learning. Proceedings of the 2021 IEEE International Conference on Blockchain and Cryptocurrency (ICBC).

[16] Eshghie M., Artho C., Gurov D. Dynamic Vulnerability Detection on Smart Contracts Using Machine Learning. Proceedings of the Evaluation and Assessment in Software Engineering (EASE 2021).

[17] Wu H., Zhang Z., Wang S., Lei Y., Lin B., Qin Y., Zhang H., Mao X. Peculiar: Smart Contract Vulnerability Detection Based on Crucial Data Flow Graph and Pre-training Techniques. Proceedings of the 2021 IEEE 32nd International Symposium on Software Reliability Engineering (ISSRE).

[18] Contract List—Ethereum Contract Library by Dedaub. https://library.dedaub.com/.

[19] GitHub yzu-wjl/SCVDIE. https://github.com/yzu-wjl/SCVDIE.

[20] Lin G., Xiao W., Zhang J., Xiang Y. (2020). Deep learning-based vulnerable function detection: A benchmark. International Conference on Information and Communications Security.

[21] Luu L., Chu D.-H., Olickel H., Saxena P., Hobor A. Making smart contracts smarter. Proceedings of the 2016 ACM SIGSAC Conference on Computer and Communications Security.

[22] GitHub ConsenSys/Mythril: Security Analysis Tool for EVM Bytecode. Supports Smart Contracts Built for Ethereum, Hedera, Quorum, Vechain, Roostock, Tron and Other EVM-Compatible Blockchains. https://github.com/ConsenSys/mythril.

[23] Mossberg M., Manzano F., Hennenfent E., Groce A., Grieco G., Feist J., Brunson T., Dinaburg A. Manticore: A user-friendly symbolic execution framework for binaries and smart contracts. Proceedings of the 2019 34th IEEE/ACM International Conference on Automated Software Engineering (ASE).

[24] Hirai Y. (2016). Formal Verification of Deed Contract in Ethereum Name Service. https://yoichihirai.com/deed.pdf.

[25] Kalra S., Goel S., Dhawan M., Sharma S. Zeus: Analyzing safety of smart contracts. Proceedings of the Network and Distributed Systems Security (NDSS) Symposium.

[26] Tsankov P., Dan A., Drachsler-Cohen D., Gervais A., Buenzli F., Vechev M. Securify: Practical security analysis of smart contracts. Proceedings of the 2018 ACM SIGSAC Conference on Computer and Communications Security.

[27] Liu Z., Qian P., Wang X., Zhu L., He Q., Ji S. (2021). Smart contract vulnerability detection: From pure neural network to interpretable graph feature and expert pattern fusion. arXiv.

[28] Huang J., Han S., You W., Shi W., Liang B., Wu J., Wu Y. (2021). Hunting vulnerable smart contracts via graph embedding based bytecode matching. IEEE Trans. Inf. Forensics Secur..

[29] Narayana K.L., Sathiyamurthy K. (2021). Automation and smart materials in detecting smart contracts vulnerabilities in blockchain using deep learning. Mater. Today Proc..

[30] Ashizawa N., Yanai N., Cruz J.P., Okamura S. Eth2Vec: Learning contract-wide code representations for vulnerability detection on ethereum smart contracts. Proceedings of the 3rd ACM International Symposium on Blockchain and Secure Critical Infrastructure.

[31] Liao J.-W., Tsai T.-T., He C.-K., Tien C.-W. Soliaudit: Smart contract vulnerability assessment based on machine learning and fuzz testing. Proceedings of the 2019 Sixth International Conference on Internet of Things: Systems, Management and Security (IOTSMS).

[32] Gensim: Topic Modelling for Humans. https://radimrehurek.com/gensim/models/word2vec.html.

[33] Gogineni A.K., Swayamjyoti S., Sahoo D., Sahu K.K., Kishore R. (2020). Multi-Class classification of vulnerabilities in Smart Contracts using AWD-LSTM, with pre-trained encoder inspired from natural language processing. IOP Sci. Notes.

[34] Polikar R. (2006). Ensemble based systems in decision making. IEEE Circuits Syst. Mag..

[35] Ren Y., Zhang L., Suganthan P.N. (2016). Ensemble classification and regression-recent developments, applications and future directions. IEEE Comput. Intell. Mag..

[36] Horowitz J.L. (2001). The bootstrap. Handbook of Econometrics.

[37] Yu X., Lu Y., Gao Q. (2021). Pipeline image diagnosis algorithm based on neural immune ensemble learning. Int. J. Press. Vessel. Pip..

[38] Yang X., Zhang Y., Lv W., Wang D. (2021). Image recognition of wind turbine blade damage based on a deep learning model with transfer learning and an ensemble learning classifier. Renew. Energy.

[39] Han D., Ding L. Financial Risk Prediction of Manufacturing Enterprises Based on SMOTE-Ensemble Learning. Proceedings of the 2021 International Conference on Management Science and Software Engineering (ICMSSE), IEEE Computer Society.

[40] Lolić I., Sorić P., Logarušić M. (2021). Economic policy uncertainty index meets ensemble learning. Comput. Econ..

[41] Zhang Y., Wang X., Han N., Zhao R. (2021). Ensemble learning based postpartum hemorrhage diagnosis for 5g remote healthcare. IEEE Access.

[42] Chen Y., Li D., Zhang X., Jin J., Shen Y. (2021). Computer aided diagnosis of thyroid nodules based on the devised small-datasets multi-view ensemble learning. Med. Image Anal..

[43] Ahakonye L.A.C., Amaizu G.C., Nwakanma C.I., Lee J.M., Kim D.-S. Enhanced Vulnerability Detection in SCADA Systems using Hyper-Parameter-Tuned Ensemble Learning. Proceedings of the 2021 International Conference on Information and Communication Technology Convergence (ICTC).

[44] Gowtham M., Pramod H.B. (2021). Semantic Query-Featured Ensemble Learning Model for SQL-Injection Attack Detection in IoT-Ecosystems. IEEE Trans. Reliab..

[45] Pennington J. GloVe: Global Vectors for Word Representation. https://nlp.stanford.edu/projects/glove/.

[46] GitHub facebookresearch/fastText: Library for Fast Text Representation and Classification. https://github.com/facebookresearch/fastText.

[47] GitHub google-research/bert: TensorFlow Code and Pre-Trained Models for BERT. https://github.com/google-research/bert.

[48] Ding Y., Suneja S., Zheng Y., Laredo J., Morari A., Kaiser G., Ray B. (2021). VELVET: A novel ensemble learning approach to automatically locate VulnErable sTatements. arXiv.

[49] He K., Zhang X., Ren S., Sun J. Deep residual learning for image recognition. Proceedings of the IEEE Conference on Computer Vision and Pattern Recognition.

